# Re-Vaccination

**Published:** 1904-03-19

**Authors:** 


The Hospital.

A Journal of

The Medical Sciences and Hospital Administration.

Vol. XXXV.—No. 912. MARCH 19, 1904.

### RE-VACCINATION.

The Bill which has been introduced into Parliament under the auspices of the Imperial Vaccination League, and of which the second reading in the House of Commons is likely, we understand, to be moved about the end of April, is said to have at least the tacit approval of the Local Government Board, but is likely, we imagine, in the present state of public affairs, to be left as an open question by the Government. The Bill provides for the compulsory re-vaccination of all children at the age of twelve or thirteen, with exception in favour of (or perhaps more properly against) the children of "conscientious objectors". The age fixed upon has reference not only to the period at which the protection of an original vaccination begins seriously to diminish, but also to the fact that among the working-classes it will in most cases intervene between the close of school life and the commencement of industry, and so become an impediment to neither. We have not heard, so far, of any powerful or organised opposition; and hence there seems some reason to hope that we may witness the inauguration of a reign of common sense in relation to an important public question from which, in time past, it has been only too rigorously excluded. As we have said, that unfortunate parliamentary creation, the conscientious objector, is to be exempted from the operation of the Bill, and his still more unfortunate children are to be left without the protection which it is intended to afford to the rest of the community. Even in their case, however, it is practically certain that an increased degree of safety will be afforded, because the liability to an epidemic of small-pox in any locality has been abundantly shown to depend, in the main, upon the proportion of susceptible persons which it contains. Obviously, therefore, the risk to the unvaccinated will be diminished by whatever set of circumstances may render the disease less likely to be prevalent in the places in which they reside. Even an ancestry of conscientious objectors would not confer upon their descendant the power of developing small-pox out of what lawyers might describe as "his own mere motion," and unaided effort.

Assuming the Bill as introduced to commend itself to a sufficient majority of the House of Commons, it will yet be subject to dangers arising from many shoals and quicksands before it can take its place upon the Statute Book. Not the least of these would arise from the probability of its being referred to the Committee on Law instead of at once to a Committee of the whole House; because the former body seems to regard it almost as a duty to make some alteration in the measures that pass under its hands, however brief and simple they may be. In the case under consideration, alterations would almost of necessity be complications, and, as such, would take from the intelligible character of the Bill as it now stands.

Although great advantages would, no doubt, be ultimately or soon secured by the passage of the Bill, we cannot but think that the Imperial Vaccination League would act more wisely if it were to extend its support to all the various subsidiary measures which may be employed for the control of small-pox prevalences. No doubt vaccination is the most complete and most trustworthy preventive; and the example of Germany shows that it may be so carried into effect as to be practically effective. But, even if the Bill should become law, many years must elapse before the population of this country will be as well protected as is that of Germany; and, even when persons of settled habitation are provided for, there will remain that curse of civilisation and that fertile source of disease, the tramp. He it is, generally speaking, who of late years has been mainly responsible for the carriage and diffusion of infection. His children, whether in infancy or at the re-vaccination period, are likely constantly to escape from the operation of the law; and he and his are a constant source of danger to the decent and respectable members of the community. If we had not the resource of vaccination, we should be compelled to deal with small-pox by the most complete possible measures of isolation and disinfection; and there seems little reason why these should not be employed, as far as possible, as adjuncts to the more radical and more effective proceeding. We fear there has been a tendency among the most convinced adherents of vaccination to disparage all subsidiary measures, perhaps for no better reason than that the anti-vaccination people have urged reliance upon these subsidiary measures alone; and we cannot but think it the best and the most enlightened policy to obtain from all sources all the security which they are calculated to afford. We have not the re-vaccination at present, and, if we obtain it, much time must elapse before we have no longer a susceptible population in our midst. Until that time arrives, it is surely better to avail ourselves of all the resources at our command than to neglect any one of them on the plea that another might be so used as to be all sufficient.

